# Efficacy of COVID-19 Booster Vaccines in Patients with Hematologic Malignancies: Experiences in a Real-World Scenario

**DOI:** 10.3390/cancers14225512

**Published:** 2022-11-09

**Authors:** Carolin Krekeler, Lea Reitnauer, Ulrike Bacher, Cyrus Khandanpour, Leander Steger, Göran Ramin Boeckel, Justine Klosner, Phil-Robin Tepasse, Marcel Kemper, Marc Tim Hennies, Rolf Mesters, Matthias Stelljes, Norbert Schmitz, Andrea Kerkhoff, Christoph Schliemann, Jan-Henrik Mikesch, Nicole Schmidt, Georg Lenz, Annalen Bleckmann, Evgenii Shumilov

**Affiliations:** 1 Department of Medicine A for Hematology, Oncology and Pneumology, University Hospital Münster, 48149 Muenster, Germany; 2 Central Hematology Laboratory, Department of Hematology, Inselspital, Bern University Hospital, University of Bern, 3010 Bern, Switzerland; 3 Department for Hematology and Oncology, University Hospital Schleswig-Holstein, 23564 Luebeck, Germany; 4 Department of Medicine B for Gastroenterology, Hepatology, Endocrinology and Clinical Infectiology, University Hospital Münster, 48149 Muenster, Germany; 5 Department of Medicine D for Nephrology and Rheumatology, University Hospital Münster, 48149 Muenster, Germany; 6 Institute of Virology, University Hospital Münster, 48149 Muenster, Germany; 7 Department of Hematology and Medical Oncology, University Medicine Göttingen (UMG), 37077 Goettingen, Germany

**Keywords:** SARS-CoV-2, hematologic malignancies, COVID-19 booster vaccines, seroconversion, Sars-CoV-2 prophylaxis

## Abstract

**Simple Summary:**

The current study provides data on the efficacy of COVID-19 prime-boost vaccines in 200 patients with hematologic and predominantly lymphoid malignancies as well as a risk-adapted guidance for the management of weak and failed responses to COVID-19 prime-boost vaccination. A total of 55% of the patients achieved seroconversion after a prime-boost vaccination. Age, lymphocytopenia, ongoing treatment and anti-CD20 B-cell depletion were independent predictors of booster failure. With each month between anti-CD20-mediated B-cell depletion and booster vaccine, the probability of seroconversion increased by 4% and serum–antibody titer levels increased by 90 AU/mL. Obinutuzumab treatment was associated with an 85% lower probability for seroconversion after prime-boost vaccination compared to rituximab. Of poor and non-responders to a prime-boost vaccine, 41% underwent a second booster vaccination and 73% underwent pre-emptive passive immunization. COVID-19 breakthrough infections were documented in only 15% patients with predominantly mild courses (93%). Next to seroconversion after prime-boost vaccination, passive immunization significantly reduced the risk of COVID-19 breakthrough infections during follow-up.

**Abstract:**

Background: Two-dose COVID-19 vaccination often results in poor humoral response rates in patients with hematologic malignancies (HMs); yet responses to COVID-19 booster vaccines and the risk of COVID-19 infection post-booster are mostly uncertain. Methods: We included 200 outpatients with HMs and predominantly lymphoid neoplasms (96%, 191/200) in our academic center and reported on the humoral responses, which were assessed by measurement of anti-spike IgG antibodies in peripheral blood as early as 14 days after mRNA-based prime-boost vaccination, as well as factors hampering booster efficacy. Previous basic (double) immunization was applied according to the local recommendations with mRNA- and/or vector-based vaccines. We also report on post-booster COVID-19 breakthrough infections that emerged in the Omicron era and the prophylaxis strategies that were applied to poor and non-responders to booster vaccines. Results: A total of 55% (110/200) of the patients achieved seroconversion (i.e., anti-spike protein IgG antibody titer > 100 AU/mL assessed in median 48 days after prime-boost vaccination) after prime-boost vaccination. Multivariable analyses revealed age, lymphocytopenia, ongoing treatment and prior anti-CD20 B-cell depletion to be independent predictors for booster failure. With each month between anti-CD20-mediated B-cell depletion and booster vaccination, the probability of seroconversion increased by approximately 4% (*p* < 0.001) and serum–antibody titer (S-AbT) levels increased by 90 AU/mL (*p* = 0.011). Notably, obinutuzumab treatment was associated with an 85% lower probability for seroconversion after prime-boost vaccination compared to rituximab (*p* = 0.002). Of poor or non-responders to prime-boost vaccination, 41% (47/114) underwent a second booster and 73% (83/114) underwent passive immunization. COVID-19 breakthrough infections were observed in 15% (29/200) of patients after prime-boost vaccination with predominantly mild courses (93%). Next to seroconversion, passive immunization was associated with a significantly lower risk of COVID-19 breakthrough infections after booster, even in vaccine non-responders (all *p* < 0.05). In a small proportion of analyzed patients with myeloid neoplasms (9/200), the seroconversion rate was higher compared to those with lymphoid ones (78% vs. 54%, accordingly), while the incidence rate of COVID-19 breakthrough infections was similar (22% vs. 14%, respectively). Following the low frequency of myeloid neoplasms in this study, the results may not be automatically applied to a larger cohort. Conclusions: Patients with HMs are at a high risk of COVID-19 booster vaccine failure; yet COVID-19 breakthrough infections after prime-boost vaccination are predominantly mild. Booster failure can likely be overcome by passive immunization, thereby providing immune protection against COVID-19 and attenuating the severity of COVID-19 courses. Further sophistication of clinical algorithms for preventing post-vaccination COVID-19 breakthrough infections is urgently needed.

## 1. Introduction

The worldwide spread of severe acute respiratory syndrome coronavirus 2 (SARS-CoV-2) since 2019 resulted in the ongoing pandemic of coronavirus disease 2019 (COVID-19), which has affected all fields of healthcare systems dramatically. Patients with hematologic malignancies are at a particularly high risk of developing severe COVID-19 and its resultant mortality due to the immunosuppressive effects of anti-cancer therapies and the underlying malignant disease itself [1,2,3,4,5,6]. The introduction of vector- and mRNA-based COVID-19 vaccines has become of particular importance in fighting the COVID-19 pandemic, resulting in up to 90–100% efficacy in the general population [7,8,9,10,11,12]. In patients with solid malignancies, COVID-19 vaccines provide almost comparable rates of seroconversion [13,14,15] and significantly decrease the risk of severe and critical COVID-19 among them [9,16]. However, patients with HMs demonstrate an impaired humoral response to both mRNA- and vector-based COVID-19 vaccines [1,13,17,18,19] and up to 50–60% [19] of HMs patients do not show any response to two vaccine doses at all [13,20,21,22,23,24]; particularly, active treatment [25] at the time-point of vaccination, lymphocytopenia [26] as well as—most emerging—prior B-cell depletion (e.g., antibodies directed against CD20 [1,22] or CD38 [21] or inhibitors of Bruton’s tyrosine kinase [BTKi] [18,19]) significantly mitigate humoral vaccine response rates. COVID-19 vaccine boosters aim to overcome waning vaccination efficacy and increase anti-SARS-CoV-2 antibody levels even in non-responders to two prior vaccines [27,28]. Hence, they are recommended for all patients with HMs independent of their previous serologic response. Nonetheless, recent studies have demonstrated about a 1.5-fold inferior efficacy of booster shots in patients with HMs compared to those with solid malignant neoplasms [29,30,31].

To date, little is known about the difference among those hematological cancer patients who respond and who do not respond to COVID-19 vaccine boosters and whom of them may be expected to benefit from the latter. The management of booster failures is another challenge in clinical routine praxis; so far, universal recommendations in such a scenario are missing and the exchange of practical experience among clinicians is of high interest in this field. Exemplarily, the application of an early second booster (4th vaccination [32]) and/or pre-emptive passive immunization (i.e., intramuscular injection of synthesized anti-COVID-19 antibodies, e.g., AZD7442 [tixagevimab/cilgavimab] [33]) might be a solution for non-responders to booster vaccines with HMs; moreover, we recently demonstrated that vaccinated cancer patients are more likely to have milder COVID-19 courses [34]. To date, to the best of our knowledge, there are no comprehensive analyses of the frequencies and severity of COVID-19 breakthrough infections after booster vaccination in patients with HMs with consideration of the individual post-booster antibody responses.

To this end, we investigated the efficacy of a prime-boost vaccination in 200 previously double-vaccinated HMs patients from our outpatient department. Subsequently, we report on the strategies that were applied as pre-emptive COVID-19 prophylaxis (e.g., passive immunization) in poor and non-responders to the vaccine. Finally, we comprehensively analyzed the severity of COVID-19 breakthrough infection courses as well as the impact of serologic immune responses following booster vaccination in this vulnerable patient collective.

## 2. Materials and Methods

### 2.1. Patients and COVID-19 Vaccination

Between December 2021 and April 2022, all 200 patients with HMs who were treated in the oncologic outpatient department of the Medical Department A for Hematology, Oncology and Pneumology, University Hospital Münster, Germany, and who received their prime-boost vaccination were retrospectively enrolled in this study. Thus, the study period was characterized by a predominance of the Omicron variant as documented in Germany since calendar week 2/2022 by the government’s central scientific institution in the field of biomedicine, the “Robert-Koch-Institute”.

The patient cohort was characterized in terms of demographics, clinical baseline data and treatment regimens. Follow-up examinations were performed according to individual physicians’ discretion and data were obtained from the treating physician.

Preceding the COVID-19 booster vaccine, all patients needed to have received two vaccine doses with either a mRNA-based (i.e., Comirnaty BNT162b2 [Biontech, Mainz Germany], Spikevax mRNA-1273 [Moderna, Cambridge, MA, USA) or vector-based vaccine (i.e., Vaxzevria AZD1222 [AstraZeneca, Cambridge, UK]) or a combination of both) according to local recommendations. The choice of the basic (double) vaccination had no influence on the inclusion of patients in this study. The response to the first two vaccine doses was not assessed prior to enrollment.

Besides the necessity of double vaccination prior to prime-booster vaccination, there were no other exclusion criteria in this study.

All patients with HMs were recommended to undergo booster vaccination (prime-booster vaccine) as early as three months after the second dose according to the general recommendation by the federal vaccination committee in Germany [Ständige Impfkommission, STIKO]. The serologic response to COVID-19 vaccines was assessed as early as 14 days after the prime-booster vaccination. A fourth vaccination or second booster vaccine was generally recommended for patients with HMs as early as early 6 months after the prime-boost vaccination. In our department, we recommended that patients with lack of seroconversion or a low serologic response (as indicated by an antibody titer ≤100 AU/mL) receive their second booster vaccine three months after prime-boost vaccination, thereby acknowledging the rapid waning titer levels [33,35,36] and the immediate re-storage of the latter after a fourth vaccination [37].

All cancer treatment modalities and all hematologic entities were considered.

The patients were analyzed with regard to (i) demographics, cancer and treatment data; (ii) seroconversion rate and antibody titer levels after a prime-boost vaccination; (iii) subsequent COVID-19 boosters and passive immunization; and finally (iv) incidence and severity of COVID-19 breakthrough infections after booster vaccination according to the WHO guidelines [38].

The retrospective data analysis was approved by the decision of the local ethics committee (Joint Ethics Committee of the Physician’s Chamber Westphalia-Lippe and the University of Münster, No. 2020-955-b-S).

### 2.2. Assessment of Humoral Response (Seroconversion) after Booster Vaccination

Anti-spike protein IgG antibodies were measured using the chemiluminescent-microparticle immunoassay (CMIA) SARS-CoV-2 IgG II Quant (Abbott, Chicago, IL, USA) in the Institute of Virology, University Hospital Münster. Serologic antibody responses were evaluated from venous blood specimens (EDTA tubes) taken during regular consultations. The SARS-COV2 IgG II Quant assay detects all IgG antibodies, including the neutralizing antibodies (nAB) that bind to a subunit of the spike protein. The detection limit is 50 arbitrary units per milliliter (AU/mL) according to the manufacturer’s manual, and the international standardized Binding Antibody Units (BAU) can be computed by a conversion factor of 7.04 (50 AU/mL corresponds to 7.1 BAU/mL). The presence of SARS-CoV-2 was confirmed by RT-PCR on nasopharyngeal or oropharyngeal swab material.

### 2.3. Definitions

Patients with hematologic malignancies under ongoing treatment (defined as cancer treatment in the last 2 months prior to prime-booster vaccination), watch-and-wait strategy or under follow-up surveillance at the time-point of booster vaccination were included in our study. Humoral response rates were determined as early as 14 days after the booster vaccination with an mRNA-based vaccine. Seroconversion was defined by the presence of anti-SARS-CoV-2 (anti-Spike) antibodies ≥ 100 AU/mL (≥14.2 BAU/mL), which corresponds to the double detection limit of the SARS-CoV-2 IgG II Quant assay (manufacturer’s definition). Values < 100 AU/mL or lacking antibody levels were defined as lack of seroconversion (booster failure). Lymphocytopenia at the time-point of booster vaccination was documented in cases where the lymphocyte count was below 1.0 × 10^9^/L. Lymphocyte subpopulations were not determined. A prime-boost vaccination was defined as the first booster after two prior COVID-19 vaccine doses, i.e., the cumulative third vaccination against SARS-CoV-2. A second booster was defined as a booster following a prime-boost vaccination. Active cancer treatment was defined as a treatment either accompanying or being completed up to 2 months prior to prime-boost vaccination.

COVID-19 breakthrough infection was diagnosed after a RT-PCR-based proof of SARS-CoV-2 during regular swabs, following a positive SARS-CoV-2 antigen test or in symptomatic patients.

### 2.4. Statistical Analysis

Categorial variables were summarized as frequencies and percentages, while continuous variables were summarized as means and standard deviations as well as confidence intervals. The Chi-square test of independence and univariable ANOVA tests were used to determine significance between prior anti-cancer treatments, patients´ characteristics, vaccination schemes and (i) seroconversion rates; as well as (ii) titer levels. A t-test for independent samples was additionally performed to determine the significance for the relationship between continuous variables (mean age, mean number of therapy lines) and seroconversion rates. For determining the significance of the relation between the proportions (i.e., achievement of seroconversion) of the population and binominal samples, such as (i) active treatment prior to vaccination and (ii) the application of prior cytotoxic and antibody treatments, the Wald test was performed. A t-test for independent samples was performed to exploit the significance between binominal samples and the antibody titer level.

Next, patients’ characteristics (age, sex), anti-neoplastic treatments, occurrence of lymphocytopenia and vaccination schedules were included in a (i) bivariable analysis or (ii) in a multivariable analysis to determine risk factors for (i) impaired seroconversion rates or (ii) detrimental antibody titer levels by linear regression, thereby assessing the regression coefficient. A linear regression for binary data was also performed to determine the significance between the time from last anti-CD20 depletion to booster vaccination and humoral response rates.

A *p*-value less than 0.05 was considered statistically significant. The reported *p*-values are two-tailed. Statistical analyses were performed with SPSS, version 28.0.1 (SPSS, Chicago, IL, USA) and JMP^®^, version 16.2.0 (Cary, NC, USA).

## 3. Results

### 3.1. Patients’ Characteristics

**Figure 1:**
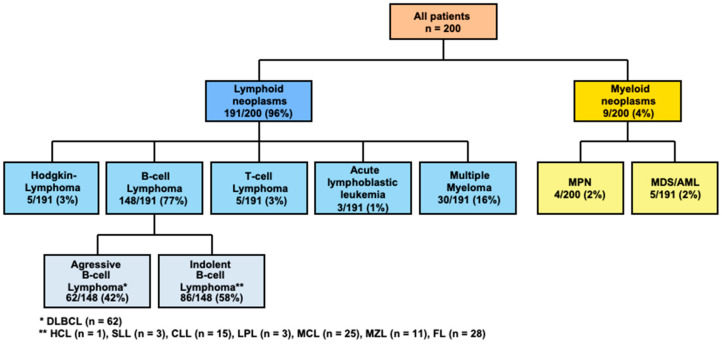
Overview of all patients and entities included in this study. DLBCL, diffuse large cell B-cell lymphoma; HCL, hairy cell leukemia; SLL, small lymphocytic lymphoma; CLL, chronic lymphocytic leukemia; LPL, lymphoplasmocytic lymphoma; MCL, mantle cell lymphoma; MZL, marginal zone lymphoma; FL, follicular lymphoma.

Patients’ demographics and disease and treatment characteristics are presented in Table 1 and Figure 1. A total of 200 patients with hematologic neoplasms who received a prime-boost COVID-19 vaccination were enrolled in the study. Prior to prime-boost vaccination, basic immunization was performed according to the local recommendations with mRNA- and/or vector-based vaccines (for further details on the distinct vaccination regimens see Supplemental Table S1).

The median age at the time-point of the booster vaccination was 65 years (range of 20–91 years) with a predominance of males (136/64). A total of 96% (*n* = 191) and 4% (*n* = 9) of patients that presented with lymphoid and myeloid neoplasia, respectively. Of the former, B-cell lymphomas were the most frequent entity (77%, *n* = 148/191) followed by multiple myeloma (16%, *n* = 30/191). Acute myeloid leukemia (AML) and high-risk myelodysplastic syndrome (MDS) (*n* = 5) were the most frequent myeloid neoplasms.

The data on COVID-19 vaccination status and its schedule are presented in Supplemental Table S1. The median time from the last cytotoxic treatment to the prime-boost vaccination was 6 months and from the last anti-CD20 treatment, if applicable, was 9 months. The presence of seroconversion and antibody titer levels were assessed at the median of 48 days after the booster vaccination.

At the time of enrollment, 47% of the patients (*n* = 94/200) were undergoing systemic cancer treatment (i.e., treatment at the time-point of booster vaccination or during the previous 8 weeks), whereas the remaining 53% were under follow-up surveillance either after systemic cancer treatment or within a watch-and-wait strategy. The details of the therapeutic modalities preceding booster vaccination are presented in Table 1.

In total, 161 patients (80%) underwent at least one lymphodepleting therapy or a therapy with high attributable risk of lymphocytopenia. Particularly, 140 patients (70%) received anti-CD20 antibodies, 23 (12%) received BTKi and 14 (7%) received anti-CD38 antibodies. A total of 19 patients (10%) received BTKi and anti-CD20 therapy successively (for more details see Table 1).

A total of 61 patients (31%) had a history of treatment with high-dose chemotherapy (HDCT) followed by autologous hematopoietic stem cell transplantation (HSCT). One of them, diagnosed with multiple myeloma, subsequently underwent allogeneic HSCT.

For details on the number of therapy lines and the remission status prior to prime-boost vaccination, see Table 1.

### 3.2. Serologic Response to COVID-19 Booster Vaccines in Study Patients

**Figure 2:**
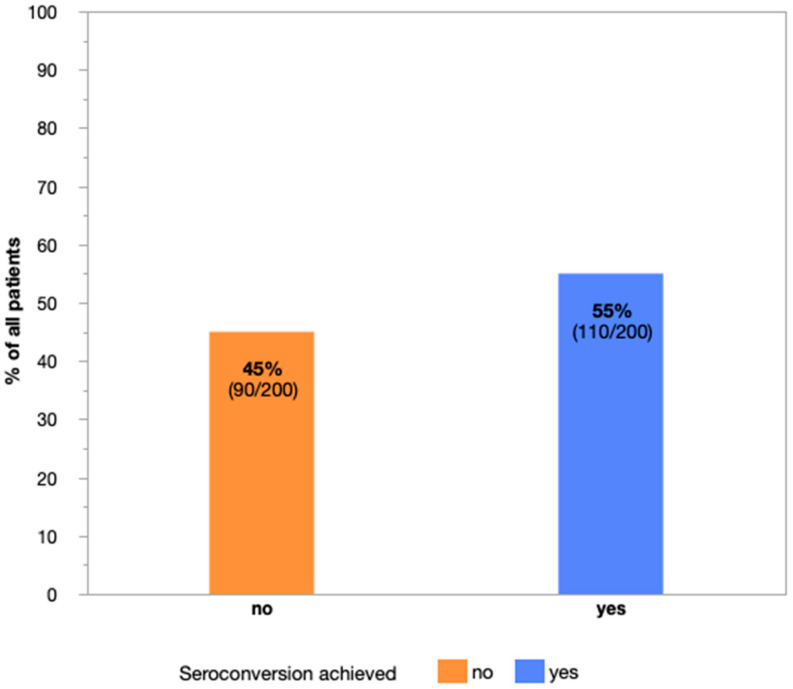
Proportion of patients with and without seroconversion (anti-spike IgG antibodies to SARS-CoV-2 ≥ 100 AU/mL) after COVID-19 prime-boost vaccination.

The incidence of serologic response versus non-response according to patients´ characteristics and treatment modalities is presented in Table 2 and Figure 2. A total of 90 patients (45%) failed to reach seroconversion even following prime-boost vaccine, whereas the remaining 110 (55%) patients presented with detectable anti-S antibodies afterwards. Among all patients, the median titer level was 258 AU/mL (range 0–40,000, mean 6982 AU/mL) and among responders it was 7052 AU/mL (range 110–40,000, mean 12,691 AU/mL), respectively.

The impact of previous cancer therapies on seroconversion and antibody titer levels and detailed information on the impact of previous cancer therapies are presented in Table 2.

The frequency of ongoing cancer treatment at the time-point of booster vaccination was significantly higher in patients who failed to reach seroconversion: 57% (51/90) without seroconversion vs. 39% (43/110) with seroconversion (*p* = 0.045). Ongoing treatment significantly mitigated the strength of humoral responses: of 94 patients, the median antibody titer value was 49 vs. 1011 AU/mL (mean values 4816 vs. 8904 AU/mL, (standard deviation [SD] 9453 vs. 14,043 AU/mL), *p* = 0.016).

Furthermore, we found an inverse relationship between the number of cancer therapy lines and a positive serological response to COVID-19 prime-boost vaccines: seroconversion rates were 57%, 25% and 0% in patients with ≤ 4, 5–6 and ≥ 7 therapeutic regimens, respectively (*p* = 0.011).

Fifty-three out of 60 patients (88%) without prior anti-CD20 B-cell depleting therapy responded to booster vaccination, while only 57 out of 140 (41%) patients previously treated with an anti-CD20 antibody did (*p* < 0.001). Patients with a history of anti-CD20-B-cell depletion had significantly lower antibody titer levels compared to patients without: median value 0 vs. 4913 AU/mL, mean value 5790 vs. 9764 AU/mL (SD 12,275 vs. 11,820 AU/mL) and *p* = 0.033. Distinct anti-CD20 antibodies affected the likelihood of humoral seroconversion differently—as far as patients receiving rituximab had higher response rates to the booster vaccination compared to those receiving obinutuzumab or their subsequent application: seroconversion rates were 49% (54/110), 14% (3/22) and 0% (0/7), respectively. Along this line, treatment with obinutuzumab was an independent negative prognostic factor for achieving seroconversion: the odds of seroconversion were reduced by 85% in comparison to rituximab (regression coefficient B −1.886, constant −0.05, Exp(B) 0.152, 95% confidence interval [0.045; 0.506], *p* = 0.002).

Likewise, patients who received a BTKi preceding booster vaccination demonstrated significantly lower antibody titers (median 17 AU/mL vs. 433 AU/mL, mean 6377 vs. 7220 AU/mL [SD 2983 vs. 7502], respectively, *p* = 0.031).

Patients (19/200) who received both BTKi and an anti-CD20 antibody prior to vaccination, applied either in combination or subsequently, had significantly lower seroconversion rates (6/19, 32% vs. 104/181, 58%, accordingly, *p* = 0.028).

Prior treatment with anti-CD38 antibodies in multiple myeloma patients (14/30 patients) did not have any impact on seroconversion rates nor antibody titer levels. All patients without prior cancer treatment or under surveillance had detectable antibodies after prime-boost vaccination (8/100; 100%).

In patients with myeloid malignancies, the seroconversion rate was 78% (7/9) compared to 54% (103/191) in patients with lymphoid malignancies. Among patients with myeloid neoplasms, the median titer level was 7556 AU/mL (mean 11,633, SD 14,996 AU/mL) compared to 177 AU/mL (6763, SD 12,106 AU/mL) in patients with lymphoid malignancies. Eight of nine patients (89%) with myeloid neoplasms underwent systemic treatment at the time-point of vaccination.

### 3.3. Impact of Time Interval between Last Cancer Treatment and Booster Vaccination

**Figure 3:**
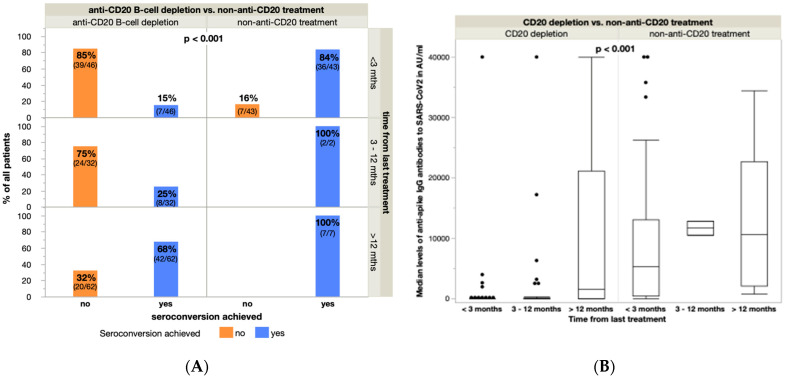
(**A**) Seroconversion rates in relation to the time from last treatment (<3 months, 3–12 months, >12 months) in the anti-CD20 B-cell depletion group vs. the conventionally treated (cytotoxic treatment, targeted treatment, non-anti-CD20 immunotherapy) group. (**B**) Median antibody titer levels in accordance with the time from last treatment compared between the anti-CD20 lymphodepleted group vs. the conventionally treated (cytotoxic treatment, targeted treatment, non-anti-CD20 immunotherapy) group. (Seroconversion rate and median antibody titer levels of patients without treatment prior to prime-boost vaccination (watch and wait-strategy, *n* = 8) are not depicted.)

We examined the impact of time interval (<3, 3–12, >12 months) between last cancer treatment and third vaccination on seroconversion rates and antibody titer levels in the anti-CD20 treated group and compared the results with the remaining, non-anti-CD20-treated patients (e.g., cytotoxic/targeted therapeutics or non-anti-CD20 immunotherapy). Anti-CD20 B-cell depletion was a negative predictor for seroconversion rates that was independent from the time between last treatment and booster vaccine (*p* < 0.001). The seroconversion rates were 15% (7/46), 25% (8/32) and 68% (42/62) in the anti-CD20 group vs. 84% (36/43), 100% (2/2) and 100% (7/7) in the non-anti-CD20 treatment group for <3 months, 3–12 months and >12 months from last treatment, accordingly (Figure 3A). The median titer levels were 0 (mean 1058, SD 5910 AU/mL), 0 (mean 2243, SD 7608 AU/mL) and 1527 AU/mL (mean 11,133, SD 15,351 AU/mL) in the anti-CD20 pre-treated group compared to 5277 (mean 9594, SD 11,772 AU/mL), 11,710 (mean 11,710, SD 1655 AU/mL) and 10,674 AU/mL (mean 14,021, SD 12,503 AU/mL) in the non-anti-CD20 treatment group (Figure 3B). With each month between anti-CD20 B-cell depletion and booster vaccination, the probability of seroconversion increased by approximately 4% (*p* < 0.001) and S-AbT levels increased by 90 AU/mL (*p* = 0.011, Supplemental Tables S2 and S3). Finally, patients with non-anti-CD20 therapies demonstrated significantly stronger humoral responses (*p* < 0.001) compared to those after anti-CD20 B-cell depletion (median titer levels 4913 vs. 0 AU/mL, mean values 9764 vs. 5790 AU/mL, SD 11,820 vs. 12,275 AU/mL).

### 3.4. Age, Prior Anti-CD20 Therapy, Ongoing Cancer Treatment and Lymphocytopenia at the Time-Point of Booster Vaccination Impede Seroconversion in Patients with Hematologic Malignancies

Subsequently, we investigated the independent predictive factors for failure of seroconversion. The data on negative predictors for impaired serologic responses in patients with hematologic malignancies are presented in Table 3.

Age was an independent predictor of a hampered serological immune response to COVID-19 booster vaccines (regression coefficient B −0.034, Exp(B) 0.966, 95% confidence interval [0.935; 0.998], *p* = 0.039). Expectedly, prior anti-CD20 therapy had the strongest detrimental effects on seroconversion; if patients had received B-cell depleting therapy, the odds for achieving seroconversion were less than a fiftieth of those without prior anti-CD20 immunotherapeutics (regression coefficient B −3.953, Exp(B) 0.019, 95% confidence interval [0.004; 0.083], *p* < 0.001). Additionally, ongoing systemic cancer treatment (regression coefficient B −1.807, Exp(B) 0.164, 95% confidence interval [0.069; 0.391], *p* < 0.001) and absolute lymphocytopenia (regression coefficient B −0.791, Exp(B) 0.453, 95% confidence interval [0.221; 0.928], *p* = 0.03) were strong independent risk factors for the lack of seroconversion. Heavily pre-treated patients tended to have a lower likelihood of seroconversion; however, this effect was not significant (regression coefficient B −0.281, Exp(B) 0.755, 95% confidence interval [0.546; 1.045], *p* = 0.091).

Prior anti-CD20 B-cell depletion and lymphocytopenia were independent negative predictors for low antibody titer levels as well. Particularly, the latter decreased by 6821 AU/mL and 4780 AU/mL (standard error 2550 and 2147 AU/mL) compared to titer levels of patients without prior B-cell depletion or a lymphocyte count above 1.0 × 10^9^/L, accordingly (all *p* < 0.03, more details are displayed in Supplemental Table S4).

### 3.5. Comprehensive Management of HMs Patients According to Serologic Response after Prime-Boost Vaccination

**Figure 4:**
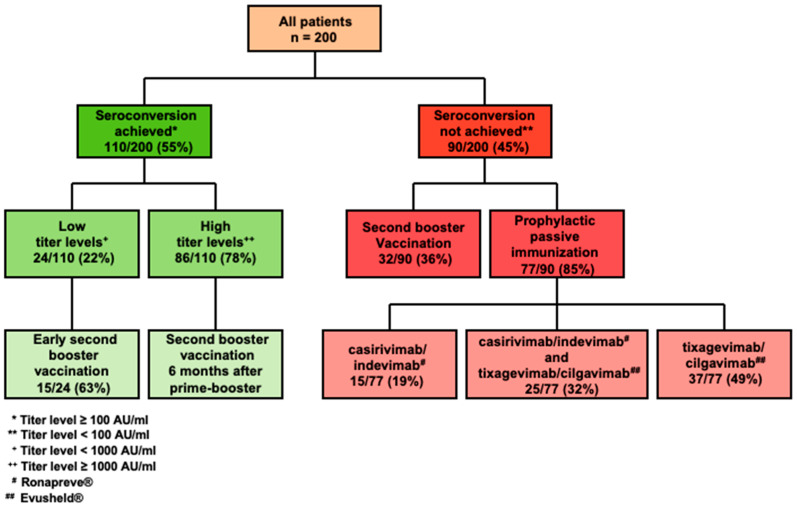
Comprehensive management of patients with hematologic malignancies according to serologic response (seroconversion) and anti-spike IgG titer levels after prime-boost vaccination.

By acknowledging (a) the impaired seroconversion rates following prime-boost vaccination in a proportion of our patients, (b) the individually variable antibody titer levels following prime-boost vaccination (range, 0–40,000 AU/mL), (c) the introduction of passive immunization and its limited availability in late 2021, and the (d) mortality risk from COVID-19, we stratified our patient cohort as indicated in Figure 4. Patients who attained seroconversion following prime-boost vaccination were subdivided in two groups according to the strength of their humoral response. Antibody titer levels < 1000 AU/mL were defined as a weak vaccine response, thereby acknowledging the rapid waning of titer levels with up to 90% diminishment 4–6 months from vaccination [35].

Twenty-two percent of all patients (24/110) who achieved seroconversion following the prime-boost vaccination were categorized as low responders (median titer level 352, mean 395, range 110–869 AU/mL) and were endorsed to receive a second booster (cumulative fourth) vaccination 3 months after the prime-booster. Of 15 patients (15/24; 68%) who subsequently received an early second booster shot, only two had a breakthrough COVID-19 infection during the further follow-up, whereas 6 of 9 patients who did not receive the second booster vaccine developed a symptomatic COVID-19 infection.

Seventy-eight percent (86/110) of the patients who achieved seroconversion following prime-boost vaccination did respond to COVID-19 vaccines with a sufficient antibody titer level ≥ 1000 AU/mL (median 10,734, mean 16,123, range 1152–40,000 AU/mL). According to the recommendation of the federal vaccination committee in Germany for patients with malignant diseases, they were suggested to receive a second booster 6 months after prime-boost vaccination. Such patients tended to have a lower probability of subsequent COVID-19 infection (0/22, 0%), whereas 9/64 (14%) patients without a second booster developed a COVID-19 breakthrough during the further follow-up; however, this effect was not statistically significant (*p* = 0.06).

### 3.6. Risk-Adjusted Pre-Emptive Prophylaxis of SARS-CoV-2 Infection in Vaccination Non-Responders with Hematologic Malignancies

All patients without seroconversion after prime-boost vaccination (90/200, 45%) were recommended to receive a second booster vaccination as early as 3 months after the primary booster. Until the data cut-off on 1 May 2022, more than a third of patients who missed seroconversion after three COVID-19 vaccines (*n* = 32/90) received a fourth (booster) vaccination.

Additionally, all patients without seroconversion after prime-boost vaccination were offered passive immunization against COVID-19. A total of 77 out of 90 patients (86%) received at least one anti-SARS-CoV-2 monoclonal antibody injection: until mid-January 2022 casirivimab/indevimab (Ronapreve^®^) was administered to 40 patients (44% of all non-seroconverted patients), followed by tixagevimab/cilgavimab (Evusheld^®^), thereby acknowledging its efficacy against the subsequently predominant Omicron variant. Finally, 62/90 patients (69%) underwent passive immunization with Evusheld^®^, and 25 of them had received Ronapreve^®^ before. It should be pointed out that passive immunization among non-responders to the third vaccination resulted in a significantly lower frequency of COVID-19 breakthrough infections during follow-up: 4/77 patients (5%) with passive immunization developed a symptomatic COVID-19 infection vs. 8/13 patients (62%) without passive immunization developed a symptomatic COVID-19 infection during the further follow-up (*p* < 0.001).

### 3.7. Disease Courses of COVID-19 Breakthrough Infections in Patients with Hematologic Malignancies after Booster Vaccination

The data on the characteristics of COVID-19-infected patients and the severity of the COVID-19 breakthrough infections following booster vaccination are presented in Table 4.

In total, 29 of 200 patients (15%), of them 27 with B-cell lymphoma, developed a RT-PCR confirmed COVID-19 breakthrough infection after at least one booster vaccination. Of those, 23 patients developed the infection after the prime-boost vaccination, whereas the 6 remaining patients (21%) had already received a second booster. A total of 40% of the respective patients (12/29) had not achieved humoral seroconversion before symptomatic COVID-19 infection, whereas the remaining patients (17/29; 60%) with symptomatic infection had achieved seroconversion. In those, the median antibody titer level was 302 AU/mL (mean 6297; range 0–40,000).

Preceding SARS-CoV-2 infection, four patients (14%) had been administered a passive immunization (three patients with casirivimab/indevimab, one add. tixagevimab/cilgavimab). There were significantly fewer patients with tixagevimab/cilgavimab applications in the symptomatic COVID-19 group compared to patients without COVID-19 breakthrough infection during the further follow-up: 1/29 (3.4%) vs. 70/171 (41%), *p* < 0.001.

Primary prophylaxis with casirivimab/indevimab (regression coefficient B −1.718, constant 0.512, Exp(B) 0.179, 95% confidence interval [0.032; 0.993], *p* = 0.049) or tixagevimab/cilgavimab (regression coefficient B −4.229, constant 0.512, Exp(B) 0.015, 95% confidence interval [0.001; 0.159], *p* < 0.001), and achievement of seroconversion after prime-boost vaccination (regression coefficient B −1.927, constant 0.512, Exp(B) 0.146, 95% confidence interval [0.30; 0.707], *p* = 0.017), were associated with a lower risk of subsequent symptomatic COVID-19 infection, while demographics, preceding treatment, the antibody titer level and the remission status did not influence the odds of COVID-19 breakthrough infection (Table 5).

The courses of COVID-19 breakthrough infection after booster vaccination were predominantly (27/29 patients, 93%) asymptomatic or mild according to the WHO stages. Only two patients (7%) had severe COVID-19 courses. Artificial respiration or high-flow oxygen therapy was not required in any patients. All patients recovered from COVID-19 infection and there were no deaths reported that were associated with SARS-CoV-2.

## 4. Discussion

The efficacy of COVID-19 boosters and the outcomes of successive COVID-19 breakthrough infections in patients with hematologic malignancies have not been thoroughly studied. Here, we comprehensively analyzed the responses to mRNA-based COVID-19 prime-boost vaccines as well as the risk factors for their failure in a large cohort of patients (*n* = 200) with various hematologic neoplasms that were treated in an outpatient setting in a large academic hospital. Furthermore, we assessed the severity of COVID-19 breakthrough infections following booster vaccination and developed an efficient, risk-adjusted strategy for the management of low or lack of serologic responses to prime-boost vaccination, thereby aiming to bridge the gap in the current guidelines and clinical practice in such scenarios.

Our patient cohort encompassed almost the entire spectrum of hematologic neoplasms and treatment modalities in an outpatient care setting. Due to an inpatient treatment in the majority of cases, patients with myeloid neoplasms were underrepresented in our study. Thus, the results of our trial cannot automatically be conferred to patients with myeloid neoplasms.

Overall, only 55% of our patients were observed with a successful seroconversion after a prime-boost vaccination (third COVID-19 vaccine). Such a low response rate is consistent with the observation of impaired humoral responses to both non-COVID-19 anti-infective vaccinations [39,40,41] and mRNA-based COVID-19 booster vaccines [29,31,42,43,44] in patients with hematologic neoplasms. In a small proportion of analyzed patients with myeloid neoplasms, the seroconversion rate was higher compared to those with lymphoid ones (78% vs. 54%, accordingly). Strong impairment of seroconversion rates after COVID-19 vaccinations in patients’ lymphoid malignancies, but not in those with myeloid malignancies, was also observed by others [45,46] and may be attributable to differences in the respective systemic treatment and risk factors, especially the lower frequency of sustained lymphodepletion in the former; however, due to the small number of patients with myeloid neoplasms in this study, these results should be interpreted carefully.

In our analysis, we revealed several factors to be associated with a lack of or poor serologic response to COVID-19 boosters. In particular, we observed lymphocytopenia at the time-point of vaccination, ongoing cancer treatment and anti-CD20 B-cell depletion therapy to be independent negative prognostic indicators of missing or weak humoral responses to COVID-19 prime-boost vaccines.

Furthermore, lymphocytopenia (<1.0 × 10^9^/L) was an independent prognostic factor of missing or low antibody titer levels in those who achieved seroconversion. These findings were in accordance with previously published data, which reported on the diminished seroconversion rates and decreased antibody titer levels in double-vaccinated hematologic patients with lymphocytopenia [26,47,48,49]. Although lymphocyte subpopulation testing was not a part of our study, we assume that lymphocytopenia was induced predominantly by B-cell deficiency—given that 38 out of 42 patients with lymphocytopenia underwent B-cell depletive therapy before.

Ongoing cancer treatment was another circumstance hindering seroconversion after COVID-19 prime-boost vaccines in patients with hematologic malignancies. Antibody titer levels were significantly lower in responders to prime-boost vaccination undergoing active anti-neoplastic treatment as compared to those with previously terminated therapy.

Following that, as well as the urgent necessity for rapid treatment in many hematologic malignancies, further identification and—if possible—avoidance of therapies with profound impact on seroconversion and strength of humoral response is a prerequisite to further protect this vulnerable collective from COVID-19 breakthrough infections. Along this line, we observed a low seroconversion rate of 43% and a significant 96% decrease of antibody titer levels in B-cell lymphoma patients treated with BTKi. The negative impact of the latter on serologic response might be attributable to the BTKi-mediated block of the B-cell receptor signaling and was comparable to the findings of others [18,19,26,50,51]. The anti-CD38 therapy results indicated a depletion of antibody-producing plasma cells and was associated with a weak or non-existent response to COVID-19 vaccines in previous trials [21,48,52]. In contrast, we did not observe any impact of prior isatuximab or daratumumab treatment on seroconversion or median antibody titer levels, although the number of patients within our cohort was low (14/200 patients, 7%).

Impaired responses to common vaccines (e.g., hepatitis B vaccination) in patients previously treated with anti-CD20 B-cell lymphodepletive therapy have already been a challenge in the pre-COVID era [6]. We elucidated the application of anti-CD20 therapy to be the strongest independent predictor of missed seroconversion after prime-boost vaccination. Additionally, the strength of the humoral response was also significantly deteriorated after the application of therapeutic regimens containing an anti-CD20 antibody (rituximab, obinutuzumab or their sequential use). Notably, in patients without prior CD20 depletion, seroconversion rates and mean titer levels were two-fold higher compared to anti-CD20 pre-treated patients. Supporting the observations of others, the negative impact of prior anti-CD20 B-cell depletion on titer and serologic response to COVID-19 vaccines was waning with time from the last treatment [1,18,19,26,53,54,55,56,57]. Indeed, patients treated with anti-CD20 antibody therapy < 12 months before prime-boost vaccination had a significantly inferior seroconversion rate of only 21% as compared to 66% among those with anti-CD20 treatment ≥ 12 months after the prime-boost vaccination (*p* < 0.001). We further elucidated that not only the time from treatment, but the distinct anti-CD20-directed drug applied influenced seroconversion rates. In fact, treatment with obinutuzumab independently reduced the odds of seroconversion by 85% in comparison to rituximab treatment (*p* = 0.002).

All five patients in our trial who were diagnosed with lymphoma and underwent CD20 B-cell depletion therapy following prime-boost vaccination presented with a median titer of 1235 AU/mL in treatment follow-up. Other authors recently outlined that while anti-CD20 therapy worsens response rates to prime-boost vaccines when given prior to vaccination, already established antibody responses were attenuated but sustained in those patients starting treatment after COVID-19 vaccination [58,59]. Hence, clinicians should weigh the necessity of an instant start to cancer treatment against the application of booster vaccinations beforehand.

A variable basic (double) immunization (i.e., vector-, mRNA- or heterologous-based double vaccination) prior to a prime-boost vaccine did not influence the odds for later seroconversion in our study.

According to our experience with COVID-19 vaccines and SARS-CoV-2 infections [3,34,60], we also provide a risk-adapted and efficient guidance for the management of weak and failed responses to COVID-19 prime-boost vaccinations. We recommended an early application of a second booster shot (as early as 3 months after the first one) for patients with minor antibody titer levels or non-responders in our academic cancer center. This practical recommendation was based on the encouraging 50% additional responses to a second COVID-19 booster vaccine (fourth vaccination) in solid organ transplant recipients in previous literature and the emerging data of additional protection rates against the prevailing Omicron variant that was enabled by the mRNA-based first-generation COVID-19 vaccines [32,61,62]. All non-responders to prime-boost COVID-19 vaccines in our university hospital were offered an ancillary prophylactic passive immunization with monoclonal antibodies (casirivimab/indevimab and/or tixagevimab/cilgavimab), which have been proven to be highly efficient towards risk reduction of a severe COVID-19 course in high-risk patients [33,63]. While patients with a second booster tended to develop COVID-19 breakthrough infections at lower frequencies, the latter were significantly diminished after passive immunization in non-responders to COVID-19 boosters. Pre-emptive monoclonal antibody injections were independent protective factors against SARS-CoV-2 infections during follow-up. A reduced risk of COVID-19 breakthrough infections in immunocompromised patients after passive immunization with tixagevimab/cilgavimab was recently also observed by others [64,65] and further outlines the importance of access to antibody administration for patients with HMs. Nevertheless, analogously to vaccine boosters, monoclonal antibodies also need to be administered repeatedly; based on pharmacokinetic/pharmacodynamic modeling data, the FDA recommends re-administration every 6 months [66].

SARS-CoV-2 infections post-COVID-19 boosters remain a challenge in clinical practice. In particular, 15% of our patients contracted SARS-CoV-2 following COVID-19 prime-boost vaccination. Although almost half of these patients lacked seroconversion post-COVID-19 boosters, the incidence of severe COVID-19 was only 7% in our cohort, which was lower in comparison to data published by others [45,67,68,69]. Accordingly, there was no COVID-19 mortality among our patients after the third, and partial (6/29 patients) fourth COVID-19 vaccine. Interestingly, other authors found that more than 50% of all hematologic patients exhibited a T-cell response against SARS-CoV-2 in an INFγ-Elispot assay of peripheral blood mononuclear cells—also being observed to be independent from prior B-cell depletion and lack of seroconversion [1,22]. Thus, considering the predominantly mild and non-fatal courses in cases of COVID-19 breakthrough infections in serologic non-responders in our cohort, booster vaccines may even be effective in B-cell lymphodepleted patients. Nevertheless, recent progress in specific COVID-19 therapies (i.e., the immediate application of sotrovimab or paxlovid^®^ after RT-PCR confirmed SARS-CoV-2 infection as done in ≈ 50% of the cases in our cohort) may have also impacted the severity of COVID-19 in our collective, thereby acknowledging the significant reduction of severe COVID-19 courses and hospitalization after its application in previous studies [70,71,72]. Furthermore, the milder courses may also be explained by the Omicron variant itself, as it causes fewer severe and critical courses and the high mortality rates of COVID-19 in HMs patients were mostly described while the Alpha and Delta variants were prevalent.

Our study has several limitations. First, most cases in our pilot study were outpatients, while only one-third of the patient cohort was heavily pre-treated (e.g., prior autologous stem cell transplantation). Second, we did not determine T-cell-mediated immunity, nor did we include it in our risk-stratified prophylactic algorithm. Furthermore, as the patients were treated in the outpatient department, lymphoid neoplasms were more frequent compared to myeloid neoplasms. Hence, the results of our trial cannot automatically be conferred to a bigger cohort of patients with myeloid neoplasms. Additionally, the mild COVID-19 courses post-booster might be attributed to the lower pathogenicity of the Omicron variant (predominant in Germany since calendar week 2/2022).

## 5. Conclusions

Overall, this study provides evidence that the humoral immune response to COVID-19 prime-boost vaccines remains significantly impaired in patients with HMs, especially in those with lymphocytopenia and ongoing cancer treatment—and particularly in those receiving anti-CD20 B-cell depletion. The distinct anti-CD20 antibody directly influences the odds for seroconversion; for example, obinutuzumab is associated with lower seroconversion rates compared to rituximab. The time from the previous cancer treatment is another crucial factor that can determine the strength of the humoral immune response. Pre-exposure prophylaxis of severe or critical COVID-19 in non-responders to COVID-19 booster vaccines is getting more sophisticated and should be administered in time, as illustrated by our study. The recent approval of an adapted bivalent vaccine targeting the Omicron subvariants BA.4 and BA.5 [73] suggests there should be further administration of booster vaccinations for patients with hematologic malignancies. We believe that our study has even more importance given that it provides a basis for the prediction of responses to upcoming booster vaccines.

## Figures and Tables

**Table 1:** Clinical characteristics and anti-cancer treatment of 200 patients with hematologic malignancies (HMs) at the time of COVID-19 prime-boost vaccination.

Parameter	Patients, *n* = 200	
Sex (M/F), *n* (%)	136/64	68%/32%
Median age, years (range)	65	20–91
**Hematologic neoplasms, *n* (%)**	***n*** = **200**	**100%**
**Lymphoma**	158	79%
*- B-cell lymphoma*	148	94%
*- T-cell lymphoma*	5	3%
*- Hodgkin lymphoma*	5	3%
**Multiple myeloma**	30	15%
**Acute leukemia**	7	3%
*- ALL*	3	43%
*- AML*	4	57%
**MPN**	4	2%
**MDS**	1	1%
**Cancer treatment preceding prime-boost vaccination, *n* (%)**		
Cytostatic (conventional) chemotherapy	165	82%
*- cytostatic agents only*	5	3%
*- combined with immunotherapy*	95	57%
*- combined with immunotherapy and targeted therapy*	25	15%
*- combined with immunotherapy and radiotherapy*	12	7%
*- combined with targeted therapy*	27	17%
*- combined with radiotherapy*	1	1%
Immunotherapy	15	8%
*- immunotherapy monoregimen*	7	47%
*- combined with radiotherapy*	8	53%
Targeted therapy	12	6%
*- targeted therapy only*	11	92%
*- combined with radiotherapy*	1	8%
No therapy yet due to first diagnosis or “watch and wait” strategy	8	4%
**Active treatment at time-point of prime-boost vaccination**	94/200	47%
**Anti-CD20 B cell-depleting therapy prior to prime-boost vaccination**	140/200	70%
*- rituximab only*	111	79%
*- obinutuzumab only*	22	16%
*- more than one CD20-depleting agent*	7	5%
**Therapy with an anti-CD38 directed antibody prior to** **prime-boost vaccination**	14/200	7%
**Therapy with BTK inhibitor prior to prime-boost vaccination**	23/200	12%
*- ibrutinib*	20	87%
*- acalabrutinib*	3	13%
**Lymphocytopenia at time-point of prime-boost vaccination**	42/200	21%
*- prior anti-CD20 B-cell depletion*	28/42	67%
*- prior BTKi therapy*	6/42	14%
*- prior therapy with anti-CD38 antibody*	4/42	9%
**Hematopoietic stem cell transplantation prior to prime-boost vaccination**	**61/200**	**31%**
*Autologous stem cell transplantation*	60	98%
*Allogeneic stem cell transplantation*	1	2%
**Number of therapy lines prior to prime-boost vaccination, *n* (%)**		
No therapy yet	7	4%
One therapy line	131	65%
Two therapy lines	31	16%
Three therapy lines	12	6%
≥ Four therapy lines	19	9%
**Remission status of HM at SARS-CoV-2 antibody testing, *n* (%)**		
Complete remission	123	61%
Partial remission	58	29%
Stable disease	5	3%
Relapsed/progressive disease	10	5%
Not yet assessed	4	2%

M, male; F, female; *n*, number; ALL, acute lymphoblastic leukemia; AML, acute myeloid leukemia; MDS, myelodysplastic syndrome; MPN, myeloproliferative neoplasm. The patient cohort was characterized according to the listed parameters in the first column. Frequencies and distributions within the cohort are displayed.

**Table 2:** Incidence of serologic response and non-response to prime-boost vaccination according to patients’ characteristics and treatment modalities.

Parameter	Seroconversion Achieved *n* = 110/200 (55%)	Seroconversion Not Achieved *N* = 90/200 (45%)	*p*-Value
Sex (M/F, in %)	66%/34%	71%/29%	0.45
Mean age, years (range)	62	67	0.08
**Therapy prior to prime-boost vaccination (%)**			
*- Cytotoxic therapy*	92%	99%	**0.045**
*- anti-CD20 B-cell depletion*	52%	91%	**<0.001**
*- BTKi*	9%	14%	0.27
*- anti-CD38 therapy*	9%	4%	0.27
*- autologous transplantation*	35%	27%	0.21
**Ongoing systemic cancer treatment at time-point of prime-boost vaccination (% of all patients. in subgroup)**	39%	57%	**0.045**
**Lymphocytopenia at time-point of prime-boost vaccination (% of patients. in subgroup)**	16%	34%	**0.03**
**Heterologous vaccination (%)**	9%	12%	0.49
**Mean therapy line** (range)	1.5 (1–6)	2.0 (1–15)	**0.01**
**Remission status prior to prime-boost vaccination**			
*- Complete Remission*	62%	61%	
*- Partial Remission*	29%	29%	
*- Stable Disease*	4%	1%	} 0.52
*- Progressive Disease*	2%	1%	
*- not yet assessed*	3%		

M, male; F, female; *n*, number. The patient cohort was characterized according to the listed parameters in the first column. Type of classification and distribution within the cohort as well as the significance level [*p* value (*t*-test)] is given for each parameter. Bold and underlined *p*-values are meant to highlight those below 0.05.

**Table 3:** Bivariable analysis of factors associated with lack of seroconversion following prime-boost vaccination in patients with hematologic malignancies.

Parameter	Coefficient (B)	EXP(B)	95% Confidence Interval of EXP(B)	*p*-Value
Constant	7.183	1316.57		<0.001
Age	−0.034	0.966	[0.935; 0.998]	**0.039**
Sex	−0.055	0.946	[0.446; 2.007]	0.885
Prior anti-CD20 B-cell depleting-therapy	−3.953	0.019	[0.004; 0.083]	**<0.001**
Prior cytotoxic treatment	−0.203	0.816	[0.06; 11.084]	0.879
Prior treatment with BTKi	−0.256	1.292	[0.409; 4.077]	0.662
Prior anti-CD38 containing therapy	−0.397	0.673	[0.902; 4.937]	0.672
Ongoing cancer treatment	−1.807	0.164	[0.069; 0.391]	**<0.001**
Heterologous vaccination	−0.336	0.715	[0.24; 2.125]	0.546
Prior autologous transplantation	−0.344	0.709	[0.301; 1.670]	0.432
Therapy line	−0.281	0.755	[0.546; 1.045]	0.091
Remission state at time point of booster vaccination	−0.008	0.992	[0.601; 1.638]	0.975
Lymphocytopenia at time point of booster vaccination	−0.791	0.453	[0.221; 0.928]	**0.03**

Patient cohort was characterized according to the listed parameters in the first column. The regression coefficient (B), the odds ratio [Exp(B)], the 95% Confidence interval as well as the significance level [*p* value (Wald´s *t*-test) is given for each parameter. Bold and underlined *p*-values are meant to highlight those < 0.05.

**Table 4:** COVID-19 courses in patients with hematologic neoplasms after prime-boost and partly second booster vaccination.

Parameter	Patients, *n* = 200	
**COVID-19 breakthrough infection after booster vaccination *n* = 29/200 (15%)**		
Sex (M/F; %)	20/9	69%/31%
Median age, years (range)	66	45–82
**B-cell lymphoma**	27	93%
MPN	2	7%
**Cancer treatment preceding COVID-19, *n* (%)**		
Conventional chemotherapy	25	86%
*- combined with immunotherapy*	11	44%
*- combined with immunotherapy and targeted therapy*	5	20%
*- combined with immunotherapy and radiotherapy*	1	4%
*- combined with targeted therapy*	8	32%
Targeted therapy	4	14%
Anti-CD20 B cell-depleting therapy prior to COVID-19	16/29	55%
Median time from last cancer treatment to COVID-19, months (range)	0.6	0–64
<3 months	15	52%
3–12 months	5	17%
>12 months	9	31%
**Remission status assessed prior to COVID-19 breakthrough infection**	29	100%
*- Complete Remission*	14	49%
*- Partial Remission*	11	38%
*- Stable Disease*	1	3%
*- Progressive Disease*	2	7%
*- not yet assessed*	1	3%
Non-responders to booster vaccination as indicated by lack of antibody titer levels prior to COVID-19	12/29	41%
Documented seroconversion after booster vaccination prior to COVID-19	17/29	59%
*- median (mean) antibody titer level (range) in AU/mL*	302 (6 298)	0–40,000 AU/ml
4th vaccination prior to COVID-19	6/29	21%
Prior passive immunization	4/29	14%
**Clinical courses of COVID-19 breakthrough infection**		
*-* asymptomatic/mild COVID-19	27	93%
*- patients with lack of seroconversion (of them with prior passive immunization)*	*10 (4)*	*37% (40%)*
*- patients with seroconversion*	*17*	*63%*
- severe COVID-19	2	7%
*- patients with lack of seroconversion (of them with prior passive immunization)*	*2 (0)*	*100% (0%)*
- critical COVID-19	0	0
**COVID-19 treatment**	15	52%
*- sotrovimab*	*10*	*67%*
*- cilgavimab/indevimab*	1	6%
*- nirmatrelvir/ritonavir*	*4*	*27%*

M, male; F, female; *n*, number; MPN, myeloproliferative neoplasm. The patient cohort was characterized according to the listed parameters in the first column. Frequencies, ranges and distributions within the cohort are displayed.

**Table 5:** Bivariable analysis of factors associated with COVID-19 after prime-booster vaccination in patients with hematologic malignancies.

**Parameter**	**Coefficient (B)**	**EXP(B)**	**95% Confidence Interval of EXP(B)**	***p*** **-Value**
Constant	0.512	1.668		0.028
Age	0.002	1.002	[0.962; 1.004]	0.912
Sex	−0.256	1.292	[0.446; 2.007]	0.885
seroconversion	−1.927	0.146	[0.030; 0.707]	**0.017**
casirivimab/indevimab	−1.718	0.179	[0.032; 0.993]	**0.049**
tixagevimab/cilgavimab	−4.229	0.015	[0.001; 0.159]	**<0.001**
active treatment at booster vaccination	0.387	1.473	[0.452; 4.803]	0.521
second booster (fourth) vaccination	−0.710	0.492	[0.155; 1.557]	0.227
Therapy line	0.268	7.37	[0.909; 1.879]	0.148
Remission state	−0.305	0.453	[0336; 0.737]	0.505
Entity	−0.190	0.827	[0.615; 1.112]	0.209
Cytotoxic therapy prior to treatment	0.215	1.24	[0.1; 15.43]	0.867
CD20 depletion prior to vaccination	−1.017	0.362	[0.101; 1.29]	0.117
Ab titer level	0.03	1	[0.972; 1.032]	0.505

Ab, antibody. Patient cohort was characterized according to listed parameters in the first column. The regression coefficient (B), the odds ratio [Exp(B)], the 95% Confidence interval as well as the significance level [*p* value (Wald´s *t*-test)] is given for each parameter. Bold and underlined *p*-values are meant to highlight those < 0.05.

## Data Availability

The data presented in this study are available on request from the corresponding author.

## Supplementary Materials

The following supporting information can be downloaded at: https://www.mdpi.com/article/10.3390/cancers14225512/s1, Table S1: COVID-19 vaccination status of the patients included in the study and time interval between last treatment and booster vaccination; Supplemental Table S2: Bivariable linear regression of association between time from last anti-CD20 therapy to booster and later seroconversion in patients treated with anti-CD20 B-cell depleting therapy (*n* = 140); Supplemental Table S3: Univariable linear regression of association between time from last anti-CD20 therapy to booster and later titer levels (in AU/mL) after booster vaccination in patients treated with anti-CD20 B-cell depleting therapy (*n* = 140); Supplemental Table S4: Multivariable linear regression of factors associated with titer levels (in AU/mL) after booster vaccination in patients with hematologic malignancies.
