# Supplementary material for: Efficacy of COVID-19 Booster Vaccines in Patients with Hematologic Malignancies: Experiences in a Real-World Scenario
Source: Cancers (Basel). 2022 Nov 9;14(22):5512. doi: 10.3390/cancers14225512 (PMC9688056; doi:10.3390/cancers14225512)
Supplement: Supplementary file 1 [file cancers-14-05512-s001.zip › cancers-2002151-supplementary.pdf]

cancers

MDPI

Supplementals

Efficacy of COVID-19 Booster Vaccines in Patients with Hematologic Malignancies: Experiences in a Real-World Scenario

Carolin Krekeler [1,*], Lea Reitnauer [1], Ulrike Bacher [2], Cyrus Khandanpour [1,3], Leander Steger [1], Göran Ramin Boeckel [4,5], Justine Klosner [1], Phil-Robin Tepasse [4], Marcel Kemper [1], Marc Tim Hennies [6], Rolf Mesters [1], Matthias Stelljes [1], Norbert Schmitz [1], Andrea Kerkhoff [1], Christoph Schliemann [1], Jan-Henrik Mikesch [1], Nicole Schmidt [7], Georg Lenz [1], Annalen Bleckmann [1] and Evgenii Shumilov [1]

Table S1. COVID-19 vaccination status of the patients included in the study and time interval between last treatment and booster vaccination.

**COVID-19 Vaccination Status**	**n of Patients**	
**All patients**	200	(100%)
**Type of first vaccination**		
Comirnaty BNT162b2 **or** Spikevax mRNA-127	177	(89%)
Vaxzevria AZD1222 **or** Johnson and Johnson Ad26.COV2.S	21	(10%)
Missing	2	(1%)
**Type of second vaccination**		
Comirnaty BNT162b2 or Spikevax mRNA-1273	191	(96%)
Vaxzevria AZD1222	7	(3%)
Missing	2	(1%)
**Type of third vaccination**		
Comirnaty BNT162b2 or Spikevax mRNA-1273	198	(99%)
Missing	2	(1%)
**Summary of COVID-19 vaccines among double vaccinated patients**		
mRNA based only	177	(89%)
Vector based vaccine followed by mRNA	14	(7%)
Vector based only	7	(4%)
Missing	2	(1%)
**Heterologous vaccination**	21	(10%)
Median time from last cytotoxic treatment until booster vaccination in months (range)	6	(0–168)
Median time from last anti-CD20 treatment to Booster vaccination in months (range)	9	(0–161)
Median time from autologous hematopoietic stem cell transplantation to booster vaccination in months (range)	23	(2–122)

Table S2. Bivariable linear regression of association between time from last anti-CD20 therapy to booster and later seroconversion in patients treated with anti-CD20 B-cell depleting therapy (n = 140).

**Parameter**	**Coefficient (B)**	**EXP(B)**	**95% Confidence Interval of EXP(B)**	***p*-Value**
Constant	-1	0.36		<0.001
Time from last anti-CD20 therapy to booster vaccination (months)	0.037	1.038	[1.016 ; 1.059]	**<0.001**

Table S3. Univariable linear regression of association between time from last anti-CD20 therapy to booster and later titer levels (in AU/mL) after booster vaccination in patients treated with anti-CD20 B-cell depleting therapy (*n* = 140).

Parameter	Coefficient (B)	95% Confidence Interval	*p*-Value
Constant	4 068	[1661; 6474]	0.001
Time from last anti-CD20 therapy to booster vaccination (months)	90	[21 ; 158]	**0.011**

Table S4. Multivariable linear regression of factors associated with titer levels (in AU/ml) after booster vaccination in patients with hematologic malignancies. BTKi, Bruton tyrosine kinase inhibitor.

Parameter	Coefficient (B)	95% Confidence Interval	*p*-Value
Constant	19071	[4507 ; 33635]	<0.001
Age	−107	[−263 ; 49]	0.178
Sex	−2588	[−6644 ; 1447]	0.206
Prior anti-CD20 B-cell depleting-therapy	−6821	[−11861; −1883]	**0.008**
Prior cytotoxic treatment	5616	[−4522; 15755]	0.275
Prior treatment with BTKi	−4109	[−9985 ; 1766]	0.169
Prior anti-CD38 containing therapy	−332	[−8573 ; 7910]	0.937
active treatment at booster vaccination	−3975	[−8484 ; 533]	0.084
Heterologous vaccination	473	[−5959; 6905]	0.885
Prior autologous transplantation	−1346	[−5853 ; 3161]	0.556
Therapy line	− 101	[−1525 ; 1726]	0.903
Remission state	−1073	[−3528 ; 1381]	0.389
Lymphopenia at time point of booster vaccination	−4780	[−9019; −540]	**0.027**
